# Intersecting Autoimmunities: ANCA and Anti‐GBM Overlap in a Patient With Sjögren’s Disease

**DOI:** 10.1155/crin/2593770

**Published:** 2026-02-27

**Authors:** Mayra Estacio, Joaquín Rodelo-Ceballos, Ligia Calderon, Alejandra Taborda-Murillo

**Affiliations:** ^1^ Facultad de Ciencias de la Salud, University of Antioquia, Medellín, Colombia, udea.edu.co; ^2^ Department of Nephrology, Hospital Universitario San Vicente Fundación, Medellín, Colombia; ^3^ Department of Pathology, University of Antioquia, Medellín, Colombia, udea.edu.co

**Keywords:** ANCA, anti-GBM, dual positivity, rapidly progressive glomerulonephritis, Sjögren’s disease

## Abstract

**Background:**

The coexistence of antineutrophil cytoplasmic antibodies (ANCAs) and antiglomerular basement membrane (anti‐GBM) antibodies defines a rare but clinically significant autoimmune overlap syndrome. This dual positivity can result in rapidly progressive glomerulonephritis, often with poor kidney outcomes. Diagnosis is particularly challenging in patients with underlying systemic autoimmune diseases, such as Sjögren’s disease, where overlapping immunopathogenic mechanisms may obscure the clinical picture. Prompt recognition and individualized treatment are critical for optimal management.

**Case Presentation:**

We describe a 62‐year‐old woman with a history of primary Sjögren’s disease who presented with acute kidney injury and urinary abnormalities. Serologic tests revealed high titers of myeloperoxidase (MPO)–ANCA and anti‐GBM antibodies, as well as ANA and anti‐Ro/La positivity. Kidney biopsy demonstrated crescentic glomerulonephritis with linear IgG deposition and significant chronic changes. Immunosuppressive therapy with high‐dose corticosteroids was initiated; however, due to advanced fibrosis and glomerulosclerosis, further immunosuppression was not pursued. The patient required dialysis but remained clinically stable during follow‐up.

**Conclusion:**

This case highlights the diagnostic and therapeutic challenges of dual ANCA and anti‐GBM antibody positivity in the context of systemic autoimmunity. In patients with autoimmune disorders such as Sjögren’s disease, a high index of suspicion is essential to detect this rare overlap, which often presents with severe kidney impairment. Although kidney prognosis is frequently poor, early identification and appropriate intervention are vital for improving clinical outcomes.

## 1. Introduction

Antiglomerular basement membrane (anti‐GBM) disease and antineutrophil cytoplasmic antibodies (ANCA)–associated vasculitis (AAV) are rare autoimmune conditions. The incidence of anti‐GBM disease ranges from 0.60 to 1.79 per million individuals annually, while AAV is estimated at approximately 17.2 cases per million person‐years [1]. Since the 1980s, dual positivity for ANCA and anti‐GBM antibodies has been recognized as a distinct clinical entity, documented across multiple cohorts worldwide [2].

Approximately 30% of patients with anti‐GBM disease also test positive for ANCA, predominantly myeloperoxidase (MPO)–ANCA, while anti‐GBM antibodies are found in about 5% of patients with AAV [3]. One proposed mechanism suggests that ANCA‐mediated neutrophil activation induces capillary injury, exposing cryptic epitopes of the glomerular basement membrane especially the NC1 domain of the α3 chain of Type IV collagen, thereby triggering anti‐GBM antibody formation [4]. Alternatively, both antibody responses may emerge in parallel due to shared genetic or environmental triggers, such as tobacco exposure or hydrocarbon inhalation [5]. A broader immune dysregulation may also be at play, particularly in patients with preexisting autoimmune diseases such as Sjögren’s disease [6]. Clinically, patients with dual positivity often exhibit overlapping features of both AAV and anti‐GBM disease. This includes severe kidney involvement and pulmonary hemorrhage typical of anti‐GBM disease, along with the relapse‐prone course more characteristic of AAV [7]. Dual positivity has been associated with worse kidney outcomes, including a higher risk of progression to end‐stage kidney disease (ESKD) [8]. Management of these patients requires aggressive early treatment to address the acute renal and pulmonary manifestations, as well as long‐term immunosuppression to prevent relapses associated with AAV. Coexisting autoimmune diseases, such as Sjögren’s disease, may complicate both the diagnostic and therapeutic landscape.

We present a case of dual MPO‐ANCA and anti‐GBM positivity in a patient with Sjögren’s disease who developed rapidly progressive glomerulonephritis, illustrating this autoimmune overlap syndrome’s clinical and histopathological complexity.

## 2. Case Presentation

A 62‐year‐old Latin American woman with a 5‐year history of primary Sjögren’s disease was referred to the nephrology department for progressive kidney dysfunction. Over the preceding 2 months, she developed fatigue, anorexia, weight loss, and a marked increase in serum creatinine (from 1.2 to 9.87 mg/dL). She denied fever, hemoptysis, edema, or gross hematuria.

The diagnosis classification of Sjögren’s disease was supported using the 2016 ACR/EULAR classification criteria. The patient had sicca symptoms and was anti‐SSA/Ro positive, which carries 3 points. Objective ocular and salivary tests and minor salivary gland biopsy results were not available from the initial diagnosis performed at an outside institution. Her medications included hydroxychloroquine and low‐dose prednisone. Physical examination revealed pallor and small joint deformities consistent with chronic inflammatory arthropathy but no edema or rash.

The laboratory test results were as follows: serum creatinine, 873 μmol/L (9.87 mg/dL); blood urea nitrogen, 105.3 mg/dL; potassium, 7 mmol/L; hemoglobin, 9.1 g/dL; and parathyroid hormone, 121.6 pg/mL. Urinalysis revealed proteinuria (3+), hematuria (2+), and dysmorphic red blood cells comprising 65% of erythrocytes in the urinary sediment. Arterial blood gases demonstrated metabolic acidosis with a pH of 7.23, HCO_3_^−^ of 13.8 mmol/L, and base excess of −12.6 mmol/L. Further immunologic testing revealed positive ANA (1:80, fine granular pattern), strongly positive anti‐SSA/Ro (> 200 U/mL) and anti‐SSB/La (125.5 U/mL), and low complement C3 (82 mg/dL). The patient tested strongly positive for MPO‐ANCA (> 100 U/mL) and for anti‐GBM antibodies (106.2 U/mL). Other infectious and autoimmune markers, including hepatitis B and C serologies, HIV, and cryoglobulins, were negative.

Kidney ultrasonography showed both kidneys of normal size and preserved corticomedullary differentiation. Chest computed tomography showed no evidence of pulmonary involvement: the lung parenchyma was normal, there were no signs of alveolar hemorrhage, no pleural or pericardial effusion, and no mediastinal or hilar lymphadenopathy.

The patient presented with a rapidly progressive glomerulonephritis, characterized by a steep decline in kidney function and urgent dialysis requirement upon admission. Hemodialysis was initiated shortly after hospitalization due to severe uremic symptoms, acidosis, hyperkalemia, and markedly elevated nitrogenous waste levels. Empiric immunosuppressive therapy was started promptly, with intravenous pulses of methylprednisolone before kidney biopsy confirmation. The patient’s ESSDAI was 19, driven predominantly by the renal domain, with minor contributions from the constitutional (low; weight loss < 5% without fever) and biological domains (low complement).

**FIGURE 1:**
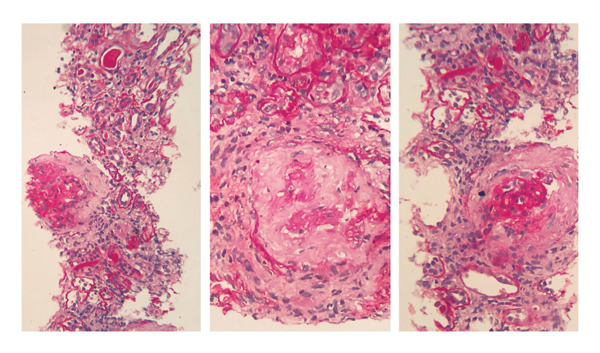
Fibrous crescents (H&E, X400)*.*

**FIGURE 2:**
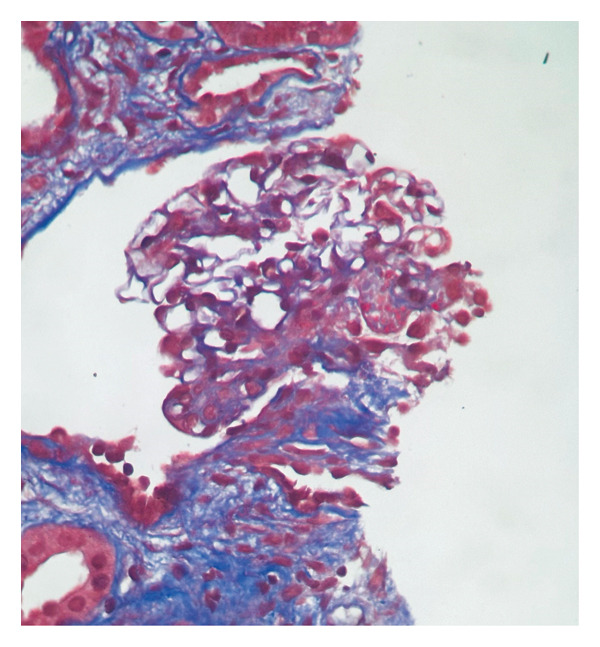
Glomerulus showed a segment of fibrinoid necrosis (Masson’s trichrome, X400)*.*

**FIGURE 3:**
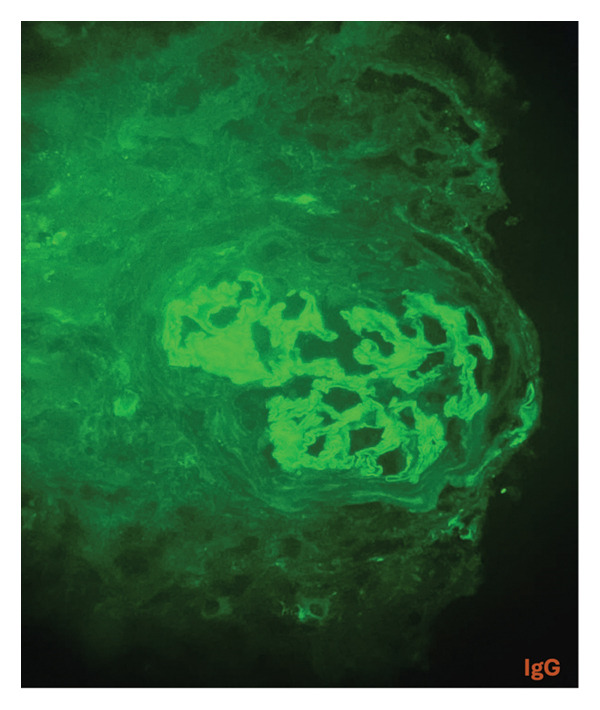
Linear positivity for IgG by immunofluorescence. The capillary walls are demarcated by a smooth, continuous line (IgG immunofluorescence, fluorescein‐labeled antihuman IgG antibody, fluorescence microscope, X400).

A kidney biopsy in light microscopy revealed corticomedullary kidney tissue with a maximum of 12 glomeruli per section. Of the total glomeruli evaluated, nine (75%) exhibited global glomerulosclerosis. Five glomeruli displayed circumferential fibrous crescents (Figure 1). One remaining viable glomerulus showed a segment of fibrinoid necrosis without endocapillary hypercellularity or epithelial crescent formation (Figure 2). No mesangial hypercellularity, capillary wall duplication, or basement membrane spikes were noted. Severe interstitial fibrosis and tubular atrophy (IFTA) affected approximately 60% of the biopsy specimen. Direct immunofluorescence showed IgG staining strongly and diffusely positive in a linear pattern along glomerular basement membranes. IgA and C1q were negative. IgM and C3 showed nonspecific trapping in areas of sclerosis. Light chains kappa and lambda showed linear capillary wall staining with strong intensity (Figure 3). The overall findings were diagnostic of necrotizing and crescentic proliferative glomerulonephritis with strong linear IgG deposition, consistent with anti‐GBM disease. The presence of MPO‐ANCA positivity and the clinical presentation supported a diagnosis of dual‐positive crescentic glomerulonephritis (anti‐GBM + AAV).

Histopathological evaluation of the renal biopsy classifies this case within the sclerotic class according to the Berden et al. classification of ANCA‐associated glomerulonephritis, which correlates with poor renal outcomes. Additionally, applying the Renal Risk Score proposed by Brix et al., which incorporates the degree of IFTA, baseline serum creatinine, and the percentage of crescents, the patient scored 16 points, placing her in the high‐risk category, also associated with a poor renal prognosis. It is important to note that both the Berden classification and the Renal Risk Score were developed for AAV, and there are currently no validated histopathological or prognostic scoring systems specifically designed for patients with dual positivity.

Chronic changes were prominent, with extensive glomerular sclerosis and severe tubulointerstitial scarring, indicating advanced disease at the time of biopsy. After multidisciplinary evaluation and in light of the extensive irreversible damage, it was determined that continued immunosuppression would not confer further benefit. Therapeutic plasma exchange was not started either due to the extensive chronic damage and the absence of pulmonary hemorrhage. The corticosteroid regimen was gradually tapered, and the patient was discharged on low‐dose prednisone with close outpatient follow‐up. Despite being dialysis dependent, she remained clinically stable.

## 3. Discussion

The case of dual positivity for both anti‐GBM antibodies and MPO‐ANCA in the setting of rapidly progressive glomerulonephritis in a patient with underlying Sjögren’s disease highlights the complex clinical challenges posed by overlapping autoimmune diseases.

Rapidly progressive glomerulonephritis is a severe form of kidney disease characterized by a rapid decline in kidney function over days to weeks. It is histopathologically classified into four major types: Type I (anti‐GBM disease), Type II (immune complex–mediated, as seen in lupus nephritis or postinfectious glomerulonephritis), Type III (pauci‐immune or ANCA‐associated), and Type IV (dual‐positive, combining features of anti‐GBM disease and AAV), the latter being a rare but increasingly recognized entity [9, 10].

It has been reported in the literature that one‐third of cases of anti‐GBM disease will have ANCA, usually MPO‐ANCA, and up to 5% of patients with AAV will have anti‐GBM antibodies [3, 7]. Serology and renal biopsy remain essential for confirming the diagnosis. Renal histopathology in patients with dual positivity for ANCA and anti‐GBM antibodies typically reveals a combination of acute and chronic lesions. Philip et al. report that most biopsies show extensive crescent formation, often involving more than half of the glomeruli, with linear IgG deposition on immunofluorescence [8]. Findings described by McAdoo et al. further support this, noting that double‐positive patients tend to exhibit more chronic injury, such as glomerulosclerosis and IFTA, compared to those with isolated anti‐GBM disease [7].

In dual‐positive patients, the levels of circulating anti‐GBM antibodies and ANCA titers can vary significantly, and their interpretation may guide both diagnosis and management. High titers of anti‐GBM antibodies are typically associated with more severe renal involvement and may correlate with the extent of glomerular injury observed on biopsy [11]. In contrast, ANCA levels, particularly MPO, are often elevated but may not directly reflect disease activity. Some studies suggest that the initial anti‐GBM titer may be a stronger predictor of short‐term renal outcome, whereas persistent ANCA positivity has been associated with a higher risk of relapse [7, 12]. In the present case, both antibodies were markedly elevated at diagnosis, consistent with the severe clinical and histopathological findings.

At the time of diagnosis, patients with dual positivity usually present with severe and rapidly progressive disease. Acute kidney injury is almost universal, with markedly elevated serum creatinine levels, often exceeding 700–800 μmol/L. Most patients require dialysis at presentation, and hematuria and proteinuria are consistently observed. Pulmonary involvement, particularly diffuse alveolar hemorrhage, is also frequent, affecting nearly half of the patients [8]. These features underscore the critical nature of early recognition and intervention in this distinct clinical entity.

Management of dual‐positive glomerulonephritis requires an aggressive, individualized approach aimed at halting immune‐mediated injury. Standard induction therapy includes high‐dose corticosteroids, cyclophosphamide, and plasma exchange, targeting both anti‐GBM and AAV components [8]. However, long‐term management must also address relapse prevention, particularly due to the AAV component, which is associated with a higher risk of recurrence. Maintenance immunosuppression is often necessary to reduce this risk.

Despite early diagnosis and aggressive therapy, the prognosis remains poor. Many patients are dialysis dependent at presentation, and only about 29% achieve kidney recovery [7]. Those who do recover often have lower anti‐GBM antibody titers at baseline. Reported 1‐year kidney survival rates range from 44% to 53%, reflecting the high burden of irreversible damage [12]. Relapse rates in dual‐positive disease are intermediate between those of isolated anti‐GBM disease, which is typically monophasic and AAV which carries a 30%–40% relapse risk, with estimates around 20%–22% at one year. One‐year mortality may reach 17%, highlighting the life‐threatening nature of this overlap syndrome [7].

Although therapeutic plasma exchange is a standard component of initial treatment for patients with anti‐GBM disease and dual‐positive vasculitis, it was not initiated in this case due to the extensive chronic damage on the biopsy and the absence of pulmonary hemorrhage. Several studies suggest limited benefit of TPE in patients with dialysis dependence and high IFTA burden at presentation [13].

The occurrence rate of anti‐GBM antibody–positive AAV in patients with Sjögren’s disease cannot be reliably estimated. A review of the literature reveals only isolated case reports. Notably, Cheng et al. reported a patient with Sjögren’s disease and rheumatoid arthritis who developed rapidly progressive glomerulonephritis with dual positivity for anti‐GBM antibodies and MPO‐ANCA. Similar to our case, renal involvement was severe and crescentic, highlighting the exceptional rarity of this overlap and supporting the notion that overlap syndrome represents an exceedingly uncommon clinical entity [6].

Renal involvement in Sjögren’s disease most commonly manifests as tubulointerstitial nephritis and, less frequently, immune‐complex glomerulopathies [14]. In contrast, this patient’s biopsy demonstrated necrotizing and crescentic GN with strong linear IgG deposition, which is characteristic of anti‐GBM disease and supports a primary anti‐GBM/AAV–driven process rather than classic SjD‐related nephropathy.

Altogether, these findings suggest that dual‐positive glomerulonephritis represents a distinct clinical entity with a particularly severe kidney prognosis and a complex therapeutic profile. This case reinforces the need for clinicians—especially nephrologists—to maintain a high index of suspicion for dual positivity in patients with RPGN, particularly those with coexisting systemic autoimmune diseases such as Sjögren’s disease. Early serologic screening, prompt kidney biopsy, and multidisciplinary management are keys to optimizing outcomes in these patients.

## 4. Conclusion

Dual positivity for anti‐GBM and ANCA antibodies represents a distinct and severe autoimmune phenotype that combines features of both anti‐GBM disease and AAV. This overlap presents significant diagnostic and therapeutic challenges, especially in patients with underlying systemic autoimmune conditions such as Sjögren’s disease. Despite timely and aggressive immunosuppressive therapy, kidney recovery is often limited, and the risk of relapse remains substantial due to the ANCA component. Early recognition, thorough serologic evaluation, and individualized treatment strategies are essential to optimize patient outcomes. Clinicians should maintain a high index of suspicion for dual positivity in patients presenting with rapidly progressive glomerulonephritis, particularly when extrarenal autoimmune manifestations are present.

## Funding

No funding was received for this study.

## Consent

Written informed consent was obtained from the patient for publication of this case report and accompanying data.

## Conflicts of Interest

The authors declare no conflicts of interest.

## Data Availability

The data that support the findings of this study are available from the corresponding author upon reasonable request.
